# Depression and brain fog as long-COVID mental health consequences: Difficult, complex and partially successful treatment of a 72-year-old patient—A case report

**DOI:** 10.3389/fpsyt.2023.1153512

**Published:** 2023-03-24

**Authors:** Maris Taube

**Affiliations:** ^1^Department of Psychiatry and Narcology, Riga Stradinš University, Riga, Latvia; ^2^Department for Depression and Crisis, Riga Center of Psychiatry and Narcology, Riga, Latvia

**Keywords:** brain fog, case report, long-COVID, depression, treatment

## Abstract

SARS-CoV-2 (COVID-19) infection can result in long-term health consequences i.e., long COVID. The clinical manifestations of long COVID include depression, anxiety, brain fog with cognitive dysfunction, memory issues, and fatigue. These delayed effects of COVID-19 occur in up to 30% of people who have had an acute case of COVID-19. In this case report, a 72-year-old, fully vaccinated patient without pre-existing somatic or mental illnesses, or other relevant risk factors was diagnosed with long COVID. Nine months following an acute COVID-19 infection, the patient's depressive symptoms improved, but memory and concentration difficulties persisted, and the patient remains unable to resume work. These long-term symptoms are possibly linked to micro-hemorrhages detected during examinations of the patient's brain following COVID-19 infection. Patient treatment was complex, and positive results were attained *via* antidepressants and non-drug therapies e.g., art, music, drama, dance and movement therapy, physiotherapy, occupational therapy, and psychotherapy.

## Introduction

The persistent and long-term effects of SARS-CoV-2 (COVID-19) infection (long COVID) may include brain fog (1, 2) consisting of cognitive dysfunction, memory issues, lack of mental clarity, forgetfulness, and fatigue, which occurs in 32% of cases; anxiety, occurring in 23% of cases; and depression, which occurs in 12% of cases (3). Long COVID has affected ~10%−30% of the people who have experienced an acute COVID-19 illness (4). The most common mental health consequences of long-COVID are fatigue, cognitive impairment, depression, and anxiety symptoms (5). While the etiology and pathogenesis of long COVID neurological syndrome (6) has not been well defined, the social consequences (such as relationship issues, fear of economic problems, and social stigma) can create additional difficulties (7).

In older people, specific pathogenic mechanisms can determine the clinical manifestations of long COVID such as hemorrhagic stroke, delirium, and long-term cognitive complaints including problems with short time memory and brain fog (8). As symptoms may persist for a long period of time, treatment may be long-lasting and require outpatient supervision (9). Lower socioeconomic status, financial insecurity, and pre-infection depression may contribute to the development of long COVID (10), and females are at higher risk for developing the condition following a mild or moderate SARS-CoV-2 infection (11). Moreover, severe cognitive symptoms and sleep disorders are also associated with females (12).

## Case presentation

A 72-year-old female experienced a severe COVID-19 infection in October of 2021 without hospitalization. She completed a vaccination course in December of 2021 and received a third (booster) dose against COVID-19. Nevertheless, in March of 2022 she had a recurrence of COVID-19, but in a milder form than the first infection. No hospitalization was needed. Following her illness, the patient attempted to return to work, however, she experienced persistent and pronounced weakness, weight loss, and difficulty concentrating. Between June 17 and July 8 2022, the patient was examined and treated in a neurological day hospital, without improvement in her health status. Next almost 2 months, she received outpatient psychiatric help and antidepressant therapy with sertraline 50 mg per day and mirtazapine 15 mg per day, without significant improvement in her condition. Inpatient psychiatric treatment was provided between August 29 and September 23, 2022 during which the patient received complex therapy—non-drug and drug treatment—as well as enteral feeding. Following hospitalization the patient visit an outpatient psychiatrist regularly (last visit January 5 2023), her condition improved and depressive symptoms decreased. However, brain fog and concentration difficulties have persisted.

### Background history

The patient has no family history of mental illnesses. During her lifetime, she has typically been energetic and active; although, she has experienced slight fluctuations in blood pressure throughout her life. She worked as a physician for 37 years until her current episode of illness. The patient is financially secure, presenting upper middle class, and has supportive relationships with her family.

### Treatment episodes

The patient was first admitted to a neurological day hospital due to generalized fatigue and weight loss. She also feared she had an oncological disease, which resulted in anxiety. Results of her examinations and treatment information are listed in Table 1. Electroencephalogram findings indicated encephalopathy, with more pronounced damage to the deep temporal structures and increased paroxysmal activity. Magnetic resonance imaging (MRI) of the head revealed diffuse and moderate enlargement of the cerebral sulci and ventricles, with multiple foci in the frontal and parietal lobes on both sides, most likely of vascular nature. There was also micro-hemorrhaging in the upper fold of the left frontal lobe. It was concluded that the patient's clinical condition was related to the effects of her previous COVID-19 infection.

**Table 1:** The results of examinations performed in an outpatient setting and a neurological day hospital.

**Date**	**Examination**	**Results**	**Comparison with previous/follow-up examinations**
06/17/2022	Magnetic resonance imaging (MRI) of the head	Diffuse moderate enlargement of the cerebral sulci and ventricles. Multiple foci in the frontal and parietal lobes on both sides, most likely of vascular nature. Micro-hemorrhage in the upper fold of the left frontal lobe.	MRI of the head (06/18/2021) performed because of complains about headache and hypochondriac thoughts. Separate, non-specific foci of vascular nature in the white matter of the cerebral hemispheres in the area of the frontal lobes frontally and subcortically. Moderate cerebral cortical atrophy, including slightly more pronounced pontocerebellar atrophy. Initial leukoencephalopathy.
06/19/2022	Electroencephalography (EEG)	Indications of encephalopathy. More pronounced damage to the deep temporal structures, and dysfunction on the left side more than on the right side, with increased paroxysmal reactivity.	EEG (10/28/2022) has no data on focality or epi-activity. EEG with increased paroxysms and reactivity changes. Marked positive dynamics compared with the EEG examination on June 2022.
12/5/2022	Transcranial doppler (TCD) of extracranial and transcranial arteries	Atherosclerotic changes in the brachiocephalic vessels without significant impact on the hemodynamics. Slightly reduced flow a. vertebralis V4 sin.	Computed tomography (CT) angiography of the brachiocephalic and intracranial vessels (04/26/2021). The internal carotid artery (ICA) dex et sin without significant changes, initial atherosclerotic changes in initial segments up to 10-20%.

As there was no improvement in the patient's condition following outpatient psychiatric treatment and the use of antidepressants, she was hospitalized in a psychiatric inpatient unit ~2 months after being discharged from the neurological day hospital. Table 2 lists the inpatient and outpatient psychiatric treatments the patient received, mental status examination by psychiatrist, as well as the results of assessments using the Clinical Global Impressions scale (CGI-S), Clinical Global Impressions scale-Improvement (CGI-I) (13), and Patient Health Questionnaire-9 (PHQ-9) (14). Upon admission to the psychiatric hospital, the patient was in a markedly depressed mood and experiencing pronounced fatigue. The patient had no energy or desire to engage in any activities. She also experienced episodic anxiety, her sleep quality had worsened, and she had poor appetite resulting in having lost 12 kg in 5 months. The patient also expressed feelings of her own impending death and of being trapped without a way out of her situation. She also had difficulty concentrating, sometimes forgetting words, with a worsening memory and a feeling of being mentally “foggy.”

**Table 2:** Hospitalization in a psychiatric inpatient unit (08/28/2022–09/23/2022): event/therapy, cognitive assessment scores (CGI-S, CGI-I, PHQ-9), treatment, and mental status evaluation.

**Date**	**Event/therapy**	**CGI-S**	**CGI-I**	**PHQ-9**	**Treatment**	**Mental status description**
08/29/2022	Hospitalization	6		21	VEN-XR until 75 mg/day/p/o, TRA at 25 mg/day/p/o, nutritional supplements, music, art, drama, dance therapy, occupational therapy, psychotherapy, physiotherapy	Markedly depressed mood, with pronounced weakness, no energy, episodic anxiety, and no desire to do anything. Patient's sleep quality has worsened. She has a poor appetite and lost 12 kg of weight in 5 months. She reports feeling like she is going to die. Patient has difficulty concentrating, sometimes forgetting words. Her memory has gotten worse and she feels mentally “foggy.”
09/23/2022	Discharge from a psychiatric hospital	3	2	8	VEN-XR 75 mg/day/p/o, TRA 25 mg/day/p/o	Patient's mood has improved, she has more energy, a more positive outlook on the future, and shows interest in her relatives. She has no anxiety. However, rapid fatigability, difficulty concentrating, unclear thinking, and forgetfulness persist. There is improved appetite, but no weight gain and her weight remains at 48 kg. Sleep quality has improved.
01/5/2023	Last outpatient follow-up visit	3	2	5	VEN-XR 75 mg/day/p/o, TRA 50 mg/day/p/o	The patient copes with daily tasks, her mood has improved, she has more energy and shows interest in her relatives. She feels better in the afternoons and evenings, but depression is worse in the mornings. The patient has no anxiety. Rapid fatigability, difficulty concentrating, unclear thinking, and forgetfulness persist. The patient eats well, but does not gain weight. She sleeps well with trazodone at a dose of 50 mg. The patient would like to return to work but does not feel able to work at the moment.

CGI-S, clinical global impressions scale; CGI-I, clinical global impressions scale-improvement; PHQ-9, patient health questionnaire-9; TRA, trazodone; VEN-XR, venlafaxine extended release.

At the hospital, the patient received medical treatment with venlafaxine up to 75 mg per day, trazodone up to 50 mg in the evening, as well as non-drug therapy (visual art, music, drama, dance and movement therapy, physiotherapy, psychotherapy, and occupational therapy) in addition to enteral feeding.

### Current status

Following her hospital discharge, the patient has continued to regularly visit a psychiatrist and takes a maintenance therapy dose of 75 mg of venlafaxine per day, and 50 mg of trazodone in the evening. The patient copes with daily tasks, her mood has improved, and she reports having more energy. In addition, she has begun to show interest in her relatives again. While depression still is worse in the morning hours she feels better in the afternoons and evenings. Furthermore, the patient no longer experiences anxiety; however, rapid fatigability, difficulty concentrating, unclear thinking, and forgetfulness persist. While her appetite has also returned, she has not gained weight and her weight remains at 48 kg. She sleeps well with 50 mg of trazodone. The patient will continue take medicines and will visit a psychiatrist monthly. The patient would like to return to work as a doctor, but does not currently feel able to.

## Discussion

Deterioration of our patient's health status with depression, anxiety, and brain fog was both temporally and clinically related to a recurrence of a COVID-19 infection. The development of long COVID was possibly related to both re-infection and the severity of infection; however, it is difficult to make unequivocal conclusions. The patient had no prior episodes of depression, and there were no adverse socioeconomic conditions that could have contributed to the development of long COVID. The patient was not hospitalized during her recurrent case of COVID-19 (which could have caused more severe consequences at a later period) and psychological stress factors were not detected (15). Vaccination did not prevent the long COVID effects of weight loss, fatigue, memory loss, brain fog, and lack of concentration (16). The patient's cognitive disorders (i.e., forgetfulness and brain fog, which persisted for 9 months after recovery from COVID-19) could have been related to the micro-hemorrhages in the upper fold of the left frontal lobe. These changes were not detected during an MRI performed because of complains about headache and hypochondria in 2021 prior to her COVID-19 illness, but were found in 2022 following the recurrence of her COVID-19 infection.

The patient's treatment was complex, with medication doses carefully selected and kept low to avoid side effects. Supplemental nutrients and non-drug therapies also played an important role, and her improvement was slow and gradual. The treatment approach for her depression was the same as for organic depression.

There are some limitations concerning the COVID-19 infection role in development of the patient's cognitive impairment. It could be independent process and beginning of dementia supported by MRI results from time period before COVID-19. Nevertheless the patient had no genetic and other important risk factors for dementia and also their functioning and performance at work as physician before COVID-19 infection was well.

## Conclusion

The development of long COVID and persistent neuropsychiatric symptoms including brain fog, memory impairment, lack of concentration, and fatigue can occur in patients without other risk factors such as hospitalization, missed vaccinations, negative psychosocial factors, or prior episodes of depression. Pathogenically, in our patient, long COVID may have been associated with the formation of micro-hemorrhages in the brain. Therapy can be partially successful using small, gradually titrated doses of antidepressants in combination with non-drug therapies.

## Data availability statement

The original contributions presented in the study are included in the article/supplementary material, further inquiries can be directed to the corresponding author.

## Ethics statement

Written informed consent was obtained from the individual(s) for the publication of any potentially identifiable images or data included in this article.

## Author contributions

MT designed the case report, gathered the data, wrote, and edited the manuscript.
